# A Brief Technique to Reduce Flashbacks of Sexual Trauma in an Adolescent: Proof-of-Concept Case Study Using Imagery Interference

**DOI:** 10.2196/79708

**Published:** 2025-11-12

**Authors:** Alex Lau-Zhu, Carmen Chan

**Affiliations:** 1 Department of Experimental Psychology, University of Oxford, Life and Mind Building, Oxford, OX1 3PS, United Kingdom, 44 1865614804; 2 Imperial College London, London, United Kingdom; 3 Oxford Health NHS Foundation Trust, Oxford, United Kingdom; 4 Linacre College, Oxford, United Kingdom

**Keywords:** adolescent, trauma, PTSD, flashback, intrusive memories, intrusions, imagery, posttraumatic stress disorder

## Abstract

**Background:**

Trauma exposure, including sexual harm, is prevalent in adolescents. A key resulting symptom relates to reexperiencing mental images of trauma, such as intrusive memories and flashbacks. Established treatments are used to address flashback memories but are hard to access, often leave remaining symptoms, and require extensive exposure to traumatic materials. An emerging approach with adult populations suggests intrusive imagery symptoms can be precisely targeted with simple cognitive tasks.

**Objective:**

We describe a first proof-of-concept demonstration of an imagery interference technique in a 15-year-old to target residual flashback symptoms after a course of treatment for posttraumatic stress disorder (PTSD) following sexual trauma, to lay the groundwork for further evaluations.

**Methods:**

A case study (Mia) was presented, drawing from routine clinical practice within the United Kingdom’s National Health Service. After 23 sessions of trauma-focused cognitive behavioral therapy, Mia received the imagery interference technique at session 24 and returned a month later for session 25 (the final session). The technique involved playing Tetris within a wider protocol informed by the science of memory malleability (eg, including brief memory recall and working memory taxation). Memory ratings (frequency, vividness, and distress) were assessed before and immediately after training on the technique and a month later. Symptoms of PTSD, anxiety, and depression were gathered at the first and final sessions. Views from Mia and her parents were also obtained.

**Results:**

For the specific flashback targeted by Mia, vividness reduced within the session (40% to 15%), distress reduced within the session (40% to 15%) and a month later (then to 10%), and frequency reduced a month later (once to zero times per week; 100% reduction). Nontargeted flashbacks also reduced in frequency (from 4 times to 1 time per month; 75% reduction). Mia described the memories as more “distant.” Symptoms of PTSD, depression, and anxiety reduced overall.

**Conclusions:**

Pending further rigorous testing beyond this single case, the imagery interference approach has potential as a low-intensity and early intervention for adolescents to address intrusive imagery of trauma, such as sexual harm, and also in other clinical contexts (eg, anxiety).

## Introduction

One in 20 young people in the United Kingdom report sexual abuse, with global data confirming higher rates in girls than boys and in older than younger teens [1-3]. A key symptom after such traumatic events is reexperiencing in the form of intrusive memories and flashbacks—persistent, distressing, and predominantly visual mental images of the trauma that pop back into mind [4-6]. They are cardinal symptoms of posttraumatic stress disorder (PTSD) and also drive distress in conditions such as anxiety and depression across the lifespan [7,8]. The highest burden of PTSD is associated with sexual traumas in youth and adult samples [9-11]. Intrusive memory symptoms can bring substantial impairment even when diagnostic criteria for PTSD are not met (eg, concentration) [7].

Established interventions for flashbacks and intrusive memories include trauma-focused cognitive behavioral therapies (CBT) and eye movement desensitization and reprocessing (EMDR) [4]. These powerful treatments aim to treat the full constellation of PTSD symptoms, rather than just flashbacks, but they also have limitations. They require extensive exposure to traumatic materials, including talking in detail about the trauma within CBT approaches. This is a key reason adolescents are often reluctant to begin treatment after trauma [12]. Residual symptoms are also common after these treatments despite meeting recovery criteria [13,14]. Many young people struggle to access these treatments (eg, due to long waiting lists) [15]. An accessible and effective intervention to specifically target intrusions may be beneficial.

A novel idea has been proposed from memory science, which can precisely address flashbacks and intrusive memories, without requiring extensive verbal recounting of trauma; for review, see [16]. This involves using a brief task (such as the shape-fitting game Tetris) to tax working memory [17] during memory malleability (ie, when a memory trace is thought to be labile and susceptible to update or interference), for example, upon retrieval [18]. This task thus interferes with capacity-limited cognitive resources needed to maintain the image-based memory, rendering it less vivid and emotional [19]. This disrupted memory is hypothesized to be re-stored [18]. The predicted effects are supported by preclinical, experimental research conducted over almost 2 decades [7,20-22]. Notably, the underpinning theory parallels a proposed explanation for the effectiveness of EMDR [23].

Initial intervention development studies have demonstrated that this imagery interference protocol can reduce the incidence of intrusive memories after real-life traumas in adults [16], regardless of the time since trauma. Critically, the approach appears to leave memory intact—an ability to recall details of the traumatic event at will seems to be retained [21,22]. This is important because professionals are often concerned about the impact of psychological therapies on trauma memory credibility within legal investigations, which frequently follow disclosures of sexual harm [24]. However, this intervention approach has yet to be translated for adolescents in experimental or clinical contexts.

Inspired by the application of this approach in adults, we present a case study as its first proof of concept for adolescents. The intervention was used predischarge to target residual flashbacks following established PTSD treatment for sexual traumas. Our aim was to gather initial information on feasibility and preliminary signal of effectiveness (ie, a reduction in the intensity and frequency of the targeted flashback) to ensure the approach is acceptable and appealing to the young person to lay the groundwork for further formal testing.

## Methods

### Case Presentation

Mia (pseudonym) is a 15-year-old adolescent girl referred to a specialist team supporting young people affected by sexual harm and their families. The referral was made by a community mental health team following an attempted overdose. At assessment, she no longer experienced suicidal intent, but reported recurring and distressing reexperiencing symptoms of an abusive relationship, including intrusive memories, flashbacks, and nightmares. She coped by pushing those thoughts away and avoiding reminders, such as music she listened to a lot during the relationship. The reexperiencing symptoms affected her self-confidence, as she continued to appraise the events as being her fault. She also reported occasional urges to self-harm by cutting, although these were generally fleeting. Her main therapy goal was to experience fewer intrusive memories and flashbacks, so that her concentration in school could be improved due to upcoming exams.

### Measures

For the specific flashback targeted as part of the imagery interference technique, vividness and distress ratings were obtained (“how vivid/distressing was the flashback?” anchored from “not at all [0%]” to “extremely [100%]”), based on prior experimental [25] and intervention protocols [26]; frequency estimates (for the prior week) were obtained retrospectively.

For the overall course of treatment, symptoms of PTSD were assessed using the Child PTSD Symptom Scale (CPSS; sessions 1, 12, 23, and 25) [27], symptoms of anxiety and depression with the Revised Child Anxiety and Depression Scale (RCADS; sessions 1 and 25) [28], and negative trauma-related appraisals were assessed with the Child Post-Traumatic Cognitions Inventory (CPTCI; sessions 1 and 25) [29].

### Intervention

#### Overview of Full Treatment

Mia received 23 one-hour sessions of trauma-focused CBT [30] over the course of 6 months. These sessions involved trauma processing by focusing on 5 key hotspots, all linked to distinct episodes of harm. She described these as highly vivid and distressing, often with strong physical sensations. She then took a 1-month break from therapy due to a busy period with exams. When she returned, she reported still experiencing some residual flashback symptoms (despite marked improvement on overall PTSD symptoms), which were the main clinical target for this report.

#### Targeting Residual Intrusion Symptoms

**Figure 1.**
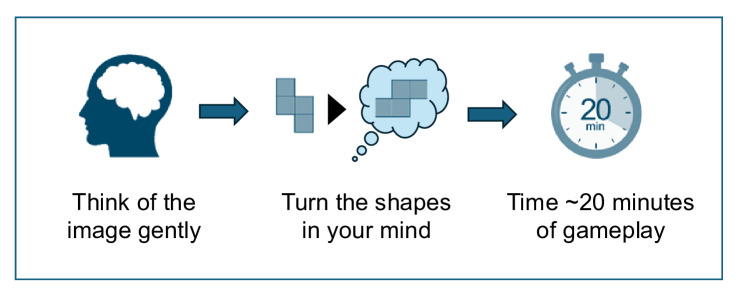
Schematic of the imagery interference technique (key steps).

The imagery interference technique (Figure 1) was delivered on session 24. Mia identified one specific hotspot memory (the specific key moment of the trauma that flashed back) that popped up in the last week and that in her view would make the most difference if it were to be addressed. She labeled this the “shock memory.” This was a mental image of a hard object approaching to hit her. She first estimated the frequency of this flashback (and others) in the last week. Then, she was instructed to gently picture this hotspot in her mind—she was specifically told to do so just for a few seconds until she had a clear picture of it in her head, but without it needing to be as emotionally intense as done during reliving sessions of trauma-focused CBT. She provided ratings of vividness and distress for this hotspot.

Afterward, the freely available game Tetris (Marathon version; Tetris Holding) was downloaded on her smartphone. The clinician (ALZ) emphasized instructions to play this by focusing on mental rotation of the upcoming shape and trying her best, rather than on performance or scores. This practice stage took around 10 minutes, and then Mia continued to play uninterrupted for another 10 minutes. Mia then provided ratings of vividness and distress again.

Mia was encouraged to use this technique on days whenever she had this flashback over the next few weeks and to apply this technique to other flashbacks she wanted to address. She could apply the technique immediately after having a flashback if it was convenient or schedule it for later within the same day, as long as the memory was briefly reactivated as part of the intervention (Figure 1). For the final session (session 25), Mia returned and estimated the frequency of the targeted flashbacks (and others) in the prior week. She also was instructed to gently picture the key hotspot in her mind as she did in the last session and provided ratings of vividness and distress. This intervention protocol delivered to an adolescent was based on prior work with adults [31,32].

### Ethical Considerations

The intervention ran as part of routine clinical care within the United Kingdom National Health Service, thus ethical approval was not needed. Parental and child consent were obtained for the study and publication. To maintain patient confidentiality, a pseudonym has been used in this report. No compensation was provided to the patient. We have abided by the Ethical Principles of Psychologists and Code of Conduct as set out by the British Association of Behavioral and Cognitive Therapies and the British Psychological Society.

## Results

### Flashback Ratings

Mia reported that the “shock memory” intruded once a week prior to the imagery interference intervention session (equivalent to 4 times in a 1-month period) and rated it as 40% vivid and 40% distressing when she gently brought it to mind as part of the first step of the intervention. She also reported flashbacks of other content, but these nontargeted ones were less frequent overall and together occurred about once a week (equivalent to 4 times in a 1-month period). Note these initial ratings were collected after finishing the preceding 23 sessions of trauma-focused CBT.

After the intervention, she pictured the memory in her mind again and rated it as 15% vivid (65% reduction) and 15% distressing (65% reduction).

A month later, at the follow-up, Mia explained that she managed to engage with the intervention 3 times the week immediately after the initial training. She reported the “shock memory” had not intruded at all in the prior week, nor in the whole period since the intervention session (100% reduction). She gently pictured the memory and rated it as 40% vivid (no reduction) and 10% distressing (75% reduction). She also reported that other flashbacks, which were not targeted in the session, intruded only once overall since the last session (75% reduction)—when this happened, it was “not powerful enough” to cause her significant disruption.

Mia also reported that she had used the technique in contexts unrelated to trauma memories. She applied it to intrusive mental images of social situations, which triggered feelings of “embarrassment.” She found that the technique reduced the emotional intensity of the image afterward. She also said she enjoyed having it as a “grounding” strategy for dealing with anxiety-provoking situations in general.

### Clinical Measures

Sessions 1‐23 corresponded to the trauma-focused CBT phase, while sessions 24‐25 (final) corresponded to the imagery interference phase.

On sessions 1, 12, and 23, scores on the CPSS were 54, 32, and 23, respectively, demonstrating a progressive decline in PTSD symptoms toward under the clinical cutoff (>31) during the CBT phase. The final score on session 25 was 22, suggesting a drop of only 1 point during the imagery interference phase.

As the imagery interference phase specifically targeted flashbacks and intrusive memories (rather than full PTSD as with the CBT phase), one relevant item was identified from the intrusion subscale of the CPSS, specific to intrusive images. Item 1 described “Having upsetting thoughts or pictures about it that came into your head when you didn’t want them to.” Before the imagery interference phase, Mia’s score on this item was 2 (referring to 2‐3 times per week or sometimes), but 1 (referring to once a week or less or a little) at the end. Although she only experienced a single flashback in the month prior (see Flashback Ratings), a score of 0 was not appropriate, as this would indicate the high threshold of “not at all” for the entire month.

Scores on the RCADS at session 1 were 63 for anxiety (below clinical cutoff) and 73 for depression (above cutoff); at session 25 (final), scores were 55 for anxiety and 56 for depression (both below cutoff). The score on the CPTCI declined from 69 at session 1 to 59 at session 25 (final), largely driven by Mia’s reduced conviction that trauma had permanently damaged the self and future.

### Qualitative Feedback

Mia said she found the imagery interference technique “helpful” for managing her remaining intrusion symptoms. A week after practicing the technique in session, she said that the memory no longer had the sense of “nowness.” Instead, she experienced more sadness, like she was “watching a TV show” as if “it happened to a friend; seeing someone I care about rather than myself.” She noted how strange it felt to be able to deliberately recall the memory without feeling overwhelmed.

Mia also described feeling able to concentrate more and experiencing a renewed sense of self-confidence. She was able to enjoy her time spent with friends and to return to her hobbies, such as playing the guitar. She said “I’m not a broken person” and “if I were to have a crash, I can get out of it,” for example, by using the technique. The gaming element of Tetris was also highlighted, as it was a “light-hearted” way to deal with difficult material and was easily accessible on her smartphone. She has also been free of self-harm for 87 days (which she was self-tracking), which had been her longest period in the last 2 years. Her parents also noted a change in Mia’s self-confidence, such as observing that she talks to adults more proactively and can comfortably make jokes.

## Discussion

### Summary of Findings

We presented the first application of an imagery interference technique to address symptoms of flashbacks and intrusive memories in the adolescence context, inspired by initial clinical demonstrations in adult populations. The intervention protocol involved playing the game Tetris on a smartphone, including brief memory recall and sustained working memory engagement, informed by memory science. Thus, the intervention is not just about playing Tetris. The clinical aim was to reduce residual reexperiencing symptoms following full PTSD treatment for sexual harm.

Our findings showed that the adolescent (Mia) found the intervention appealing and was willing to try it; she reported changes in key properties of flashback memories (including frequency and distress) within the intervention session and at a 1-month follow up, with subjective accounts of perceived benefits. Overall improvements in symptoms of PTSD, depression, anxiety, trauma-related appraisals, and self-harm were also observed in relation to the full therapeutic arc, which included preceding trauma-focused CBT prior to introducing the imagery interference technique.

### Clinical Implications

These preliminary adolescent data align with emerging laboratory [21,22] and clinical findings with adults [16]. The brief and precise nature of the protocol meant that minimal adaptations to the adolescent context were needed, especially as it does not require detailed discussions of trauma (eg, sexual abuse). The game- and phone-based nature of the intervention made it particularly accessible to the adolescent.

As this case illustrated, the aim of the technique was not to replace an established treatment approach, which we used as first-line treatment (ie, trauma-focused CBT). A relatively simple technique could provide an additional tool for managing distressing symptoms, such as residual ones common after trauma-focused treatments [13,14]. Given problems with access in child and adolescent mental health services [15], the technique presented here holds potential as an early intervention; for example, while young people are waiting for treatment or in the form of a scalable, single-session intervention [33]. Many adolescents are often reluctant to initiate trauma-focused therapies [12]; thus, this technique may also facilitate a gentler initial engagement with trauma memory.

Although clinical guidelines recommend CBT—often delivered by highly-trained therapists—within the first 3 months after trauma [34], many young people’s first professional contact after disclosures of sexual harm is with nonspecialists, such as teachers and social workers. Interventions that can be more easily deployed have potential for scalability, including for high-risk and low-resource contexts. However, despite the seemingly simple nature of the technique, appropriate training and supervision may still be important as with other low-intensity interventions [35]. A game-based technique like this may also be harnessed as an adjunct within established treatment for out-of-session practice, given the frequent challenges with therapy homework with adolescents [36].

### Limitations

The changes observed may not be solely attributed to the imagery interference technique. Other explanations for the observed effects on intrusive memories should be considered (eg, a continuation of improvement after 23 sessions of CBT). The single case design was limited by the routine setup, in collaboration with the adolescent, rather than designed as a research study. Building on these initial data supporting acceptability, stronger designs for causal inference should be used in the future, such as single-case experimental designs, which can better account for time trends prior to introducing the intervention.

The reduction in intrusive memory indices was not easily captured by the CPSS, an established questionnaire for PTSD symptoms. However, this only captured intrusive memories with a single item, which may not be sufficiently sensitive. Novel measurement approaches to capture changes in intrusive memories could be considered, such as diary methods and validated scales.

### Conclusions

Early intrusive symptoms are important to ameliorate to support key developmental tasks, such as school learning. Our preliminary data lay the groundwork to evaluate the use of this imagery interference technique in adolescents, so we can better attribute any observed benefits to the technique itself rather than other therapeutic elements. Beyond sexual harm, the benefits on flashbacks and intrusions are predicted to extend to other types of trauma experienced by young people, as the focus is on shared memory mechanisms. Finally, as Mia used the technique for addressing anxiety, where intrusive imagery is also prominent [8], the technique merits additional explorations in a range of clinical contexts relevant to youth mental health.

## Abbreviations

CBT: cognitive behavioral therapy
CPSS: Child PTSD Symptom Scale
CPTCI: Child Post-Traumatic Cognitions Inventory
EMDR: eye movement desensitization and reprocessing
PTSD: posttraumatic stress disorder
RCADS: Revised Children's Anxiety and Depression Scale
